# A New Method to Quantify and Compare the Multiple Components of Fitness—A Study Case with Kelp Niche Partition by Divergent Microstage Adaptations to Temperature

**DOI:** 10.1371/journal.pone.0119670

**Published:** 2015-03-30

**Authors:** Vasco M. N. C. S. Vieira, Luz Valeria Oppliger, Aschwin H. Engelen, Juan A. Correa

**Affiliations:** 1 MARETEC, Instituto Superior Técnico, Universidade Técnica de Lisboa, Av. Rovisco Pais, 1049-001, Lisboa, Portugal; 2 Center of Applied Ecology and Sustainability, Facultad de Ciencias Biologicas, Pontificia Universidad Católica de Chile, Santiago, Chile; 3 CCMAR—Centre of Marine Sciences, University of Algarve, Gambelas, 8005-139 Faro, Portugal; University of Toronto, CANADA

## Abstract

**Point 1:**

Management of crops, commercialized or protected species, plagues or life-cycle evolution are subjects requiring comparisons among different demographic strategies. The simpler methods fail in relating changes in vital rates with changes in population viability whereas more complex methods lack accuracy by neglecting interactions among vital rates.

**Point 2:**

The difference between the fitness (evaluated by the population growth rate λ) of two alternative demographies is decomposed into the contributions of the differences between the pair-wised vital rates and their interactions. This is achieved through a full Taylor expansion (i.e. remainder = 0) of the demographic model. The significance of each term is determined by permutation tests under the null hypothesis that all demographies come from the same pool.

**Point 3:**

An example is given with periodic demographic matrices of the microscopic haploid phase of two kelp cryptic species observed to partition their niche occupation along the Chilean coast. The method provided clear and synthetic results showing conditional differentiation of reproduction is an important driver for their differences in fitness along the latitudinal temperature gradient. But it also demonstrated that interactions among vital rates cannot be neglected as they compose a significant part of the differences between demographies.

**Point 4:**

This method allows researchers to access the effects of multiple effective changes in a life-cycle from only two experiments. Evolutionists can determine with confidence the effective causes for changes in fitness whereas population managers can determine best strategies from simpler experimental designs.

## Introduction

There are several reasons justifying comparison between demographies and their responses to the environment. The geographical distribution of species and their abundance is often constrained by physiological limitations [1, 2, 3, 4, 5, 6] associated to environmental variables; temperature being considered a determinant factor with a significant impact on survival, reproduction and/or growth [7]. This has often been observed in benthic marine macroalgae [8, 9, 10, 11, 12, 13]. Then, it is relevant to compare the different demographic responses of populations of the same species subject to different environments. But species may also be constrained by biological barriers. Total niche overlap being impossible, competitors tend to differentiate their adaptation to the physical environment which may result in a spatial or temporal mutual exclusion with local dominance by the fitter competitor. Then, it is relevant to compare the different demographic responses of populations of both competitors subject to the same environment. In both cases it is necessary understanding how environmental factors affect the vital rates and ultimately the fitness of marine organisms [3, 14]. In order to do that, the multiple fitness components across the entire life cycle need be quantitatively estimated [15, 16].

Several methods can be used to compare demographic performances. The mathematically simplest solutions involve univariate comparisons i.e. each vital rate separately, which poses many drawbacks: (1) too many repeated statistical analysis, (2) inexistent power to synthesize information and (3) inability to detect interactions among vital rates. Oppliger et al [13] used the observed values for each vital rate disabling comparing and ranking them according to relevance. Furthermore, the biggest drawback is differences among vital rates alone do not justify niche differentiation. A fitness differentiation still needs to be proved. The clearest example is probably that of post-reproductive mammals where, whatever the change in their survival rates, it has absolutely no impact in their population’s success [17]. The slightly more sophisticated solutions of sensitivity and elasticity analyses [17], also called prospective analysis [18], calculate the potential of vital rates to induce changes in the population growth rate (λ). However, this does not decompose an effective change in λ into its several sources i.e, effective changes in the vital rates. Consider the vital rate promoting the largely higher elasticity of λ remaining constant from population ‘a’ to population ‘b’. Then, it has absolutely no part in the divergent adaptation of these populations to the environment. For that to happen it is also required an effective change (i.e. not just potential change) in the vital rates from population ‘a’ to population ‘b’. To comply with this three alternatives have been presented. The perturbation analysis (Caswell [17] pp 557–560), which is only valid for density dependent models with populations at equilibrium (i.e, λ = 1), and the retrospective analysis [18] ignore potentially important interactions (2^nd^ and higher order terms), thus rendering the analysis inaccurate. Life-Table Response Experiments (LTRE), as proposed by Caswell [17], may also account for 2^nd^ order terms but neglect higher-orders that can be fundamental in time-variant models (i.e, seasonal environments or sequences of developmental stages) often described by periodic demographic models where the life cycle viability is particularly dependent on specific sequences of vital rates. To accurately determine the relevance of each sequence it is required to estimate its partial derivatives to an order as high as the number of parameters within. The LTRE presentation by Caswell [17] does not specify how to estimate such derivatives. The quantification of every term required, thus providing the analysis with absolute precision, is achievable with a full Taylor expansion of the original model and presently demonstrated applied to a periodic matrix model of Kelp microstages. Taylor series expansion is one of the most famous and widespread tools of calculus being applied everywhere from physics, to statistics, to every branch of engineering [19, 20, 21]. Nevertheless, only rarely it has been used in population dynamics [17, 22] and ecological modelling [23]. Attached to the full Taylor expansion we propose a numerical solution for the calculus of all its related partial derivatives. The demography of microstages of two *Lessonia sp* species were used as study case. These were already studied in the univariate based analysis by Oppliger et al [13], thus allowing an objective evaluation of the gains brought by this new method.

Kelps may occur from polar to inter-tropical zones but are typically more abundant in cold temperate waters. Thermal responses for the kelp macroscopic stage [9, 24] and the microscopic stages [8, 13, 25, 26, 27, 28, 29, 30] are generally consistent with their geographical distributions. The intertidal kelp, formerly *Lessonia nigrescens* Bory, habiting the Chilean coast was recently found to consist of two cryptic species: the ‘Northern’ *Lessonia berteroana* ranging between 16°S and 30°S and the ‘Southern’ *Lessonia spicata* ranging between 29°S and 41°S [31, 32]. These two species are reproductively isolated [33] and were never found co-existing in the same location, even within their transition zone where a mosaic of pure populations was observed whatever the spatial scale investigated [13, 31, 34]. With such contrasting geographical distributions both species experience diverging environmental conditions, particularly when it comes to water temperature with the northern species occurring in warmer waters than the southern species. They are also differentially exposed to environmental disturbances and instabilities. During the El Niño event of 1982/83, massive mortalities affected the northernmost populations. Still, some survived possibly as the result of local adaptation to high temperatures [35, 36]. Presently, it is intended to determine how the microstages of these two cryptic species are differentiating their physiological and demographic responses to temperature with an effective impact on niche occupation.

## Materials and Methods

### Experimental design

Fertile sporophytes were sampled in eight intertidal locations along the Chilean coast with especial sampling effort upon the overlapping transition zone of the two cryptic species. The remaining locations were considered as central populations. Sampling sites were Iquique (IQ: 20°25’62”S 70°12’48”W), Carrizal Bajo (CA: 28°04’2”S 71°08’36”W), Chañaral de Aceituno (AC: 29°04’03”S 71°29´26”W), Choros Ventana (CH: 29°12’57”S 71°28’23”W), Coquimbo-Cruz (CO: 29°57’15”S 71°42’05”W), Río Limarí (LI: 30°44’10”S 71°42’05”W), Las Cruces (LC: 33°30’09”S 71°38’01”W) and Valdivia (VA: 39°46’75”S 73°23’49”). No special permissions were required to collect species in any of those areas, and neither kelp species are protected or endangered. 25 years data (1982–2007) from the Advanced Very-High Resolution Radiometer (AVHRR) satellite (Casey and Cornillon 1999) showed Sea Surface Temperatures (SST) ranged from 11.0°C (monthly mean minima) to 15.2°C (monthly mean maxima) in Valdivia and from 16.2°C to 23.0°C in Iquique. Temperature in the transition zone ranged from 13.2°C to 17.9°C. The two *Lessonia sp* are isomorphic and thus were distinguished based on length polymorphism of the atp8 ⁄ trnS mitochondrial marker [31, 33]. The fertile sporophytes from each location had spore release induced and cultures initiated in 50mL Falcon tubes (BD Biosciences, San Jose, CA, USA). The experimental design included 5 temperature treatments applied to 8 locations, resulting in a total of 40 demographies, each composed of 3 replicates with 2 pseudo-replicate tubes. The temperature treatments had three fixed temperature conditions (10°C, 15°C and 20°C) and two variable temperature regimes (10–15°C and 15–20°C) where the culture temperature was changed every three days. Inside the cultured tubes seven microscopic stages were defined following Sauvageau [37]: S1) settled meiospores, S2) germinated spores, S3) gametophytes of 1–2 cells, S4) gametophytes of > 2 cells, S5) reproductive female gametophytes, i.e. bearing oogonia, S6) fertilised female gametophytes, i.e. bearing microscopic sporophyte, and S7) male gametophytes. The abundances of these microstages were recorded at days 2, 5, 13, 17 and 24.

### The periodic matrix model

All individuals were spores (*S*_*1*_) at the start of the experiment (*t*_*0*_). The population vector at day *t* (**n**_**t**_) was calculated by left multiplying the previous population vector (**n**_**t-x**_) by the demographic matrix for the respective day (**P**_**t**_). Thus, the population vectors at the end of the experiments were estimated by:

$$n_{24}=P_{24}\times P_{17}\times P_{13}\times P_{5}\times P_{2}\times n_{0}$$ (1)

The microstages’ development was described by the sequence of matrices (Equation 2). Be aware that *p*_*i*,*j*_ in **P**_**x**_ is not identical to *p*_*i*,*j*_ in **P**_**y**_. At the end of the experiment not all individuals of all population×temperature combinations had reached stage *S*_*6*_. Optimally the experiment should proceed until there is none individual in any of the modelled stages. To overcome this and model the fate of the remaining individuals, three consecutive 7day bins with dynamics equal to the average **P**_**24**_ were assumed and named **P**_**#**_. The surviving *S*_*6*_ would have reproduced yielding spores (*S*_*1*_), thus closing the cycle. As the sporophyte dynamics were black boxes, a constant fertility from stage *S*_*6*_ back to *S*_*1*_ was assumed (**P**_**f**_). The population growth rate (*λ*) was calculated by Equation (3). It was a measure for the fitness of each demography i.e. a population incubated at a given temperature.

$$P_{2}=\left[\begin{array}{cc} S_{1}\\ S_{1} & p_{1,1}\\ S_{2} & p_{2,1} \end{array}\right]\;P_{5}=\left[\begin{array}{ccc} S_{1} & S_{2}\\ S_{1} & p_{1,1} & 0\\ S_{2} & p_{2,1} & p_{2,2} \end{array}\right]\;P_{13}=\left[\begin{array}{ccc} S_{1} & S_{2}\\ S_{3} & 0 & p_{3,2}\\ S_{4} & 0 & p_{4,2}\\ S_{5} & 0 & p_{5,2}\\ S_{6} & 0 & p_{6,2} \end{array}\right]\;P_{17}=\left[\begin{array}{ccccc} S_{3} & S_{4} & S_{5} & S_{6}\\ S_{3} & p_{3,3} & 0 & 0 & 0\\ S_{4} & p_{4,3} & p_{4,4} & 0 & 0\\ S_{5} & p_{5,3} & p_{5,4} & p_{5,5} & 0\\ S_{6} & p_{6,3} & p_{6,4} & p_{6,5} & p_{6,6} \end{array}\right]\;P_{24}=\left[\begin{array}{ccccc} S_{3} & S_{4} & S_{5} & S_{6}\\ S_{3} & p_{3,3} & 0 & 0 & 0\\ S_{4} & p_{4,3} & p_{4,4} & 0 & 0\\ S_{5} & p_{5,3} & p_{5,4} & p_{5,5} & 0\\ S_{6} & p_{6,3} & p_{6,4} & p_{6,5} & p_{6,6} \end{array}\right]\;P_{f}=\left[\begin{array}{ccccc} S_{3} & S_{4} & S_{5} & S_{6}\\ S_{1} & 0 & 0 & 0 & f_{6} \end{array}\right]$$ (2)

$$\lambda =\frac{n_{f}}{n_{0}}=P_{f}\times P_{\#}^{3}\times P_{24}\times P_{17}\times P_{13}\times P_{5}\times P_{2}$$ (3)

### Taylor expansion analysis

The analysis started by setting a reference demography (**P**_**a**_) with its λ_*a*_ and an alternative demography (**P**_**b**_) with its λ_*b*_ = λ_*a*_+Δλ. The difference Δλ, representing an absolute change in fitness, was decomposed into the effects of each vital rate or sequence of vital rates applying a Taylor expansion (Equation 4), where *h*_*i*_ = Δ*x*_*i*_ = *x*_*b*,*i*_-*x*_*a*,*i*_. Regularly, Taylor expansions have a zero order term (*n* = 0). In this case it corresponded to λ_*a*_, which had to be subtracted. Thus, the formulation was simplified by starting the sum from *n* = 1. In demographic models there is no ‘dead’ stage (except for Markov chain analysis) and thus all transitions give positive contributions to λ. Implicitly, all partial derivatives are positive and thus any term of the Taylor expansion could only be negative when its related *h*_*i*_ was negative i.e, when a transition had a lower probability in the alternative demography than in the reference demography. Hence, a positive term represented investment in a transition by the alternative demography whereas a negative term represented discard.

$$\Delta_{\lambda }=\lambda_{b}-\lambda_{a}=\sum_{n=1}^{\theta }\frac{1}{n!}\left[{\left(h_{1}\frac{\partial }{\partial x_{1}}+h_{2}\frac{\partial }{\partial x_{2}}+\ldots +h_{i}\frac{\partial }{\partial x_{i}}\right)}^{n}.\lambda_{a}\right]$$ (4)

To estimate the Taylor expansion each entry *p*_*i*,*j*_ in each matrix **P**_**d**_ was considered separately. For a matter of simplicity, parameter *p*_*i*,*j*_ is presently referred to as *x*_*i*_. However, the *i* and *j* subscripts are unrelated: in *p*_*i*,*j*_ they refer to the row and column coordinates whereas in *x*_*i*_ it refers to the rank of the vital rates when sorted from first (1) to last (θ). Notice that Equation (3) could be reformulated stating λ as a function of these entries, that is λ = *f*(*x*_1_,*x*_2_,…,*x*_*i*_): the left product of the matrices extensively written would give a single yet extremely long equation in the form of sums of terms. Each of the terms was the product of 9 elements, each element from a distinct matrix. Therefore, the Taylor expansion would have been full (i.e: remainder = 0) at the 9^th^ order terms (*n* = 9); however, **P**_**f**_ and *f*_*6*_ were constants and thus the Taylor expansion was already full at the 8^th^ order terms (*n* = 8). Also, **P**_**#,a**_ and **P**_**#,b**_ were equal as both were given by the average **P**_**#**_. The rationale has not having any *h*_*i*_ relative to **P**_**#**_ overestimating the importance of the related vital rates. Then, the Taylor expansion was full at the 5^th^ order terms (*n* = 5). So, overall every term of Δλ was in the form of Equation (5) with *k*_*i*_≤1, *n* = ∑*k*_*i*_ and *n*≤5.

$$\left(\frac{n!}{\prod k_{i}!}\right)\prod h_{i}^{k_{i}}\left(\frac{\partial^{n}\lambda }{\prod \partial x_{i}^{k_{i}}}\right)|\begin{array}{c} k_{i}\leq 1\\ n=\sum k_{i} \end{array}=n!\prod h_{i}^{k_{i}}\left(\frac{\partial^{n}\lambda }{\prod \partial x_{i}^{k_{i}}}\right)|\begin{array}{c} k_{i}\leq 1\\ n=\sum k_{i} \end{array}$$ (5)

The ∂*λ*/∂*x*_*i*_ were numerically estimated calculating the left product of all the matrices with the amendment that the matrix where *x*_*i*_ occurred had all its elements turned to 0 except *x*_*i*_ that was turned to 1. Similarly, ∂^*n*^ *λ*/(∂*x*_*1*_∂*x*_*2*_…∂*x*_*i*_) was obtained calculating the left product of all the matrices with the amendment that the ones where any of the *x*_*i*_ occurred had all their elements turned to 0 except the *x*_*i*_ which were turned to 1. As an example, ∂^*2*^ *λ*/(∂*P*_2_ *p*_1,1_∂*P*_13_ *p*_3,2_) was estimated from Equation (6) and its related term in Δλ was estimated by expression (7). Remember λ is a sum of sequences (of nine vital rates) and, from the basic rules of differentiation, each sequence can be independently partially derived. In the example given, setting every other vital rate to zero eliminates all sequences where either *P*_2_ *p*_1,1_ or *P*_13_ *p*_3,2_ are absent, as it should be because the derivative of a constant is zero. All remaining sequences are of the form *f*_6_×*p*_6,*j*_×*p*_*i*,*j*_×*p*_*i*,*j*_×*p*_*i*,*j*_×*p*_*i*,*j*_×*p*_3,2_×*p*_2,1_×*p*_1,1_, with 3≤*i*,*j*≤6 because there is an extensive ramification of possible paths in matrices *P*_17_ and *P*_24_. From the basic rules of differentiation, the second order partial derivative of any of these sequences to *P*_2_ *p*_1,1_ and *P*_13_ *p*_3,2_ can be obtained setting these specific vital rates to 1, thus yielding *f*_6_×*p*_6,*j*_×*p*_*i*,j_×*p*_*i*,*j*_×*p*_*i*,*j*_×*p*_*i*,*j×*_ *p*_2,1_. Also, remember *f*_6_ was arbitrarily chosen to close the loop. Therefore, it is always the same constant whereas the possible effect of its change is besides this analysis.

$$\frac{\partial^{2}\lambda }{\partial P_{2}p_{1,1}\partial P_{13}p_{3,2}}=P_{f}\times P_{\#}{}^{3}\times P_{24}\times P_{17}\times {P_{13}|}_{\begin{array}{l} p_{3,2}=1\\ p_{i,j}=0 \end{array}}\times P_{5}\times {P_{2}|}_{\begin{array}{l} p_{1,1}=1\\ p_{i,j}=0 \end{array}}$$ (6)

$$2!h_{P_{2}p_{1,1}}h_{P_{13}p_{3,2}}\frac{\partial^{2}\lambda }{\partial P_{2}p_{1,1}\partial P_{13}p_{3,2}}$$ (7)

In the end there could be close to 5000 terms most of them relative to specific combinations of vital rates (as is the case of Equation 7). Some of these combinations could be particularly interesting. Nevertheless, it was not practical to infer about all of them and the same is expected to occur with most future applications of this method. The best alternative was to evenly partition the 2^nd^ and higher order terms of Δλ among its constituent vital rates after Δλ was fully decomposed. In the example of Equation 7, the term was split in half with each half going either to *P*_2*p*1,1_ or *P*_13*p*3,2_.

### Significance estimates for the Taylor expansion terms

Some components of Δλ were big and most probably reflected deterministic change whereas others were small and most probably reflected only stochasticity. Thus, the significance of each term was estimated developing a custom made permutation test. The null hypothesis was all demographies came from the same pool. Hence, vital rates did not change significantly between any two demographies implying their related Δλ component was not significantly different from zero. To simulate the null hypothesis *n* pairs **P**_**b**_-**P**_**a**_ were randomly generated, with *n* = 1000. Each vital rate in **P**_**a**_ and **P**_**b**_ was randomly chosen from the original set of 40 observed demographies with their transitions. For each **P**_**b**_-**P**_**a**_ pair was performed the Taylor expansion as explained above, leading to empirical distributions (with n elements) of each Δλ component (as estimated from Equation 5) under the null hypothesis. The alternative hypothesis was not all demographies came from the same pool. Hence, for at least some of the vital rates, these did change significantly between two specific demographies as also did their related Δλ components. Each of these was compared with its related empirical distribution under the null hypothesis. Comparison was done based on the absolute values (equivalent to a two tail comparison) as the terms could be positive or negative.

### Multivariate analysis of the Taylor expansion terms

With 40 demographies (8 locations each incubated at 5 temperatures regimes) there were 40×39/2 = 780 possible pairwise combinations (**P**_**b**_-**P**_**a**_). Furthermore, there were 28 vital rates appearing in at least one of the 40 demographies. Such vast data sets need be synthesized to generate useful information. We propose comparing each demography (i.e. each specific population×temperature) with the average demography and subjecting the results to a Principal Components Analysis (PCA). In this case there were 40 observations (the demographies) and 28 variables (the Δλ terms relative to each vital rate). In most ecological applications of PCA variables are measuring different things, come in different units and present widely different variances. To overcome this problem and not overrate some variables while underrating others, these PCAs are based in correlation matrices, corresponding to standardizing variables to have zero mean and unit variances. In this work all variables were measuring the same thing i.e. Δλ and it was fundamental to preserve their original values. Therefore, it was performed a PCA based on the covariance matrix as it preserved the original variances and thus the original Δλ. The PCA procedures were following Vieira [38]. These were preliminary results used for the first step of a non-orthogonal (Promax) factor analysis. In the second step, the obtained factors were examined and compared with the previous principal components. From the application of the selection algorithms presented in Vieira [38] (again, n = 10000 and α = 0.05) resulted in pratical terms in the retention of only the factor loadings with an absolute value bigger than 0.4 while the remaining were set to 0, which enabled identifying the demographic pathways preferred by the demographies. These scores were different from Δλ. In fact, negative scores could also be obtained from positive Δλ in factors with negative loadings. A second analysis estimated the overall fitness gain from each demographic pathway summing their significant Δλ components and comparing among demographies by means of permutation tests (n = 10000 and α = 0.05).

## Results

**Fig 1:**
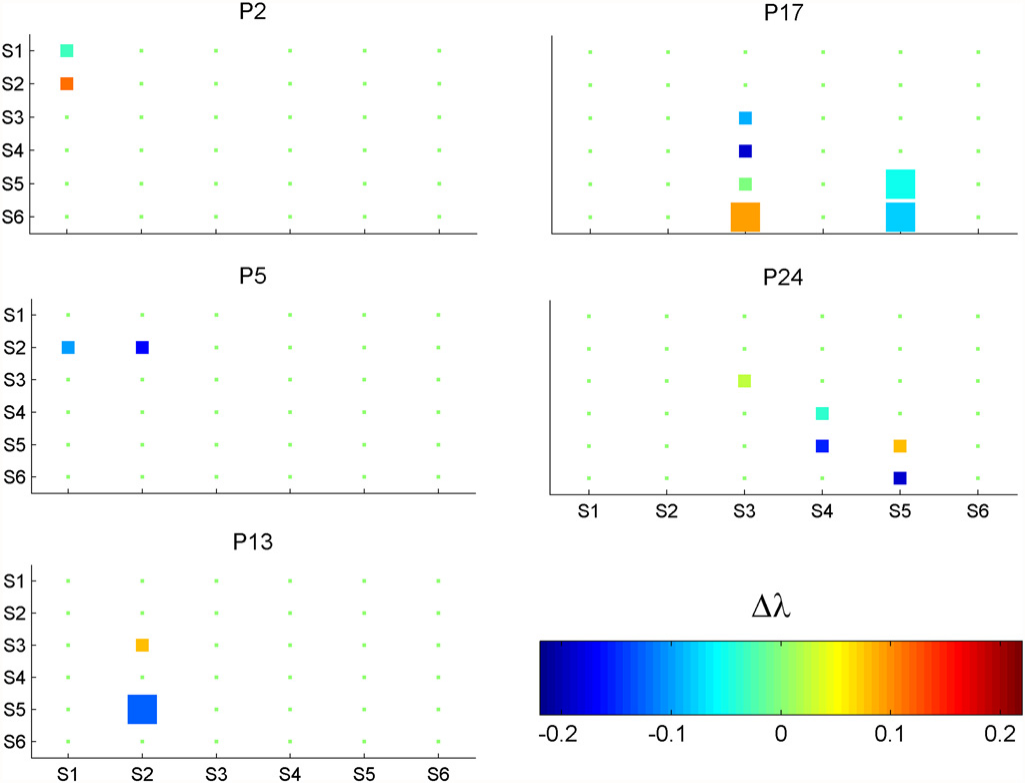
Decomposition of the change in the population growth rate (λ) from the Va population (P_a_) to the Iq population (P_b_) both incubated at 10°C. Each square is the Δλ term (λ_*b*,*i*_-λ_*a*,*i*_) relative to the respective periodic matrix parameter. Therefore, positive represents advantage to P_b_ whereas negative represents advantage for P_a_. The significant terms are in bigger squares.

The simpler data analysis was exemplified comparing the northern most (Iquique being P_b_) to the southern most (Valdivia being P_a_) population when incubated at 10°C (Fig. 1). With these settings a positive Δλ term represented advantage for the Iquique demography whereas a negative term represented advantage for Valdivia. Conspicuous patterns emerged illustrating what can be expected from future applications. Iquique spores germinated faster but Valdivia spores although germinating slower still survived. Therefore, P_2_p_21_ was positive whereas P_2_p_11_ was negative. However, this difference was not significant. Valdivia females maturated faster: from the 5^th^ to the 13^th^ day Valdivia exhibited more germinated spores developing to mature females whereas Iquique spores tended to stay as 1–2 celled gametophytes. Thus the negative P_13_p_52_ and the positive P_13_p_32_. As Iquique females maturated slower there were more of them reaching the mature and fertilized stages later at the 17^th^ day. Thus the positive P_17_p_52_ and P_17_p_62_ terms. However, as more Valdivia females were already mature on the 13^th^ day more survived as so or got fertilized to the 17^th^ day. Hence the negative P_17_p_55_ and P_17_p_65_ terms. All terms representing slower development still brought some advantage to its population because those individuals still had the chance to reproduce. Otherwise, these terms should be zero. However, the true values of these terms are uncertain as the demographies had to be assumed after the 24^th^ day, demonstrating the importance of following the fate of every individual until its end. Counting only the significant Δλ terms, Valdivia females maturated faster and more of these younger females survived as so or were later fertilized. Hence the significantly negative P_13_p_52_, P_17_p_55_ and P_17_p_65_. Although maturating slower Iquique females were still fertilized. Hence the significantly positive P_17_p_63_. Overall, the significant terms summed to 0.1175–0.1538–0.095–0.0588 = -0.19, representing a large advantage for the Valdivia population (southern species) when incubated at 10°C due to enhanced sexual maturation and fertilization.

The higher order terms had a fundamental role in the accuracy of these results. In the 10°C test, summing the absolute values of the Δλ terms, 1^st^ order terms summed to 0.1701, 2^nd^ order terms to 0.2621, 3^rd^ order terms to 0.2038, 4^th^ order terms to 0.0665 and 5^th^ order terms to 0.0074. Overall, 1^st^ order terms represented 23.96% of the Taylor expansion, 2^nd^ order terms represented 36.92% and higher order terms the remaining 39.12%. However, positive and negative terms cancel out. Summing their real values the 1^st^ order terms represented 16.21% of the Taylor expansion and higher order terms the remaining 83.79%. In the 20°C test, summing the absolute values the 1^st^ order terms represented 48.66% of the Taylor expansion and higher order terms the remaining 51.34% whereas summing their real values 1^st^ order terms represented 67.07% and higher order terms the remaining 32.93% of the Taylor expansion.

**Fig 2:**
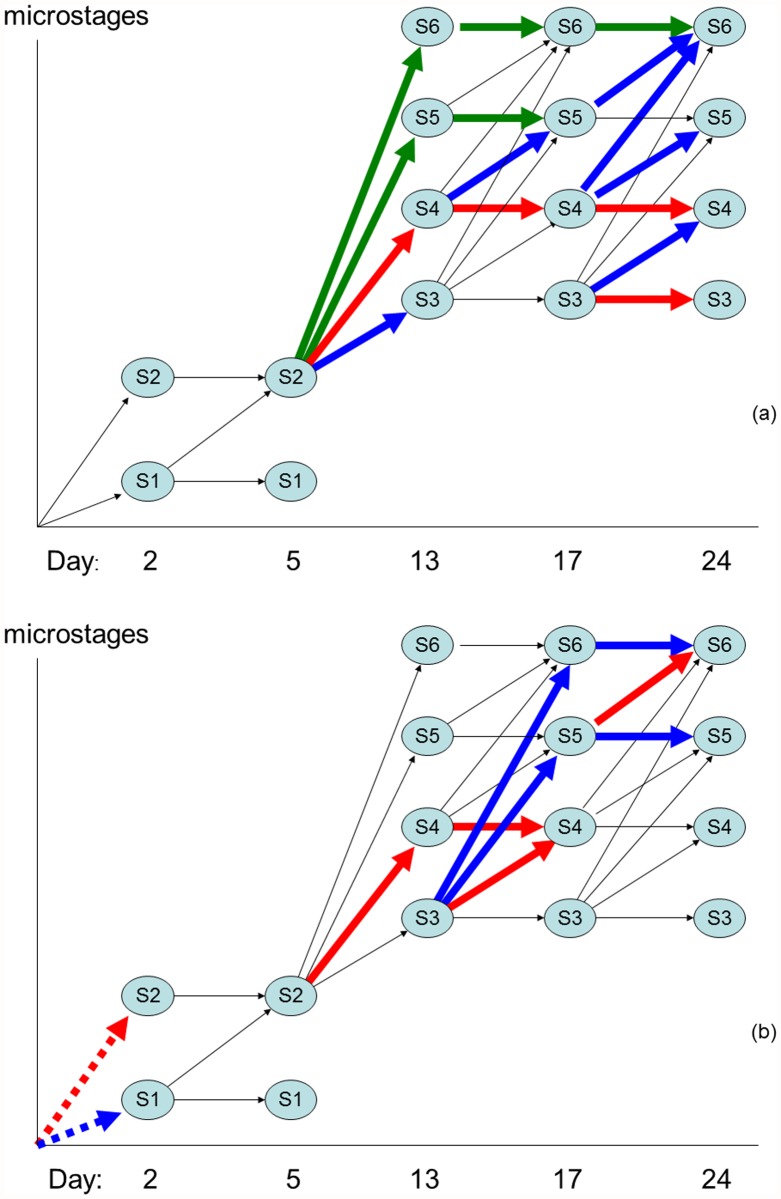
Contrast in the microstage dynamics described by (a) the two largest factors: vital rates belonging to factor 1, all with positive loadings (green); factor 2, with positive loadings (blue) and with negative loadings (red); and (b) factors 3 and 4: (solid line) factor 3, (dotted line) factor 4, (blue) positive loadings and (red) negative loadings.

Comparing all the demographies required the numerically more complex data analysis, still better able to synthesize information. The principle component analysis (PCA) identified four significant factors of which the bigger three largely overlapped variables. The Promax rotation of these four factors justified 42.35% variation (the communality) when accounting only for the loadings with an absolute value bigger than 0.4, having identified demographic pathway differentiations among temperatures and populations. The largest three factors, aggregating 13.69%, 12.13% and 10.28% of communality, differentiated among the effect of temperature (p_1_ = 0.0002, p_2_ = 0.0001 and p_3_ = 0.0354) on microstages growth from germinated spores (S2) developing into reproductive (S5) and fertilised (S6) female gametophytes (Fig. 2). Only the third factor also differentiated among populations (p_1_ = 0.7499, p_2_ = 0.9727 and p_3_ = 0.0044). Populations at 15°C demonstrated a tendency for fast reproduction (Fig. 2a green arrows, post hoc p≤0.0002). Populations incubated at 10°C or 10–15°C demonstrated a tendency for slower reproduction (Fig. 2a,b blue arrows), still faster than populations incubated at 20°C or 15–20°C (post hoc p<0.0127) in which the development mainly stagnated in the S4 stage relative to non-reproductive gametophytes with more than 2 cells (Fig. 2 red arrows). The fourth largest factor aggregated 6.24% of communality describing a contrast between demographies where within the first two days spores only settled or also germinated (Fig. 2b) depending on population (p = 0.0109) but not on temperature (p = 0.0659). These results were severely biased by the mixed temperatures incubations. Removing these from the analysis and differences were no longer significant among populations (p = 0.2285) but were now significant among temperatures (p = 0.034) with the 10°C and 15°C incubations significantly preferring different pathways compared to 20°C incubations (blue versus red transitions in Fig. 2b).

**Fig 3:**
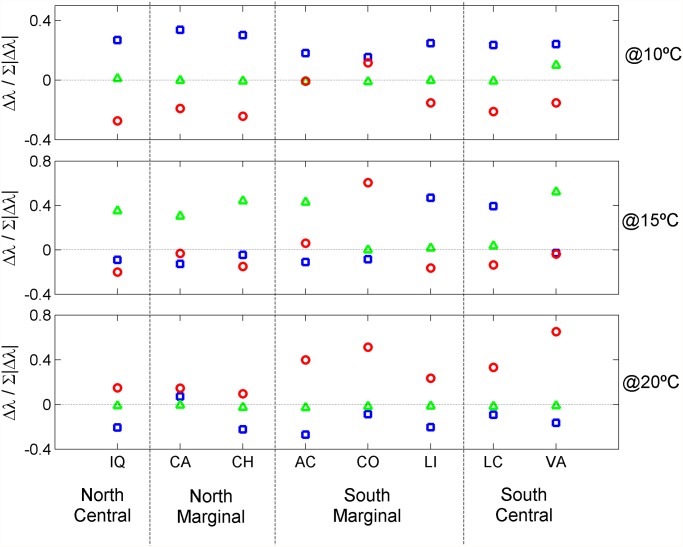
Preference of each population to follow the blue, green or red path (as defined in Fig. 2) depending on the incubation temperature.

For each population incubated at a fixed temperature all the Δλ relative to each pathway were standardized, summed and compared (Fig. 3). At 10°C populations slowed reproduction (blue pathway) but did not stagnate (red pathway). At 15°C five populations reproduced faster (green pathway) discarding the slow and stagnant pathways. This was common to north and south, central and marginal populations. However, two south populations preferred the slow reproduction pathway while another south population tended to stagnate. At 20°C particularly the southern populations preferred the stagnant pathway. Following this pathway did not represent a true advantage as such individuals had difficulties maturating and many stood infertile for the whole 24 days experiment, their fate being assumed afterwards. Such artificialization allowed them to maturate, reproduce and thus contribute to λ artificially increasing the fitness of the stagnant pathway. The correct form to interpret these results is warmer temperatures (20°C) severely decreased the southern species ability to sexually maturate whereas the northern species was little affected.

## Discussion

The Taylor expansion analysis showed that sexual maturation and fertilization of female gametophytes are the key aspects of the kelp microstages differentiation between the two *Lessonia sp* cryptic species whereas spore settlement and germination is irrelevant. Therefore, the niche partition they display along a latitudinal temperature gradient is set by their diverging fertility components of fitness and nothing else, at least within the microstages’ demography. These conclusions, contrasting from those by Oppliger et al [13], were driven from results obtained with less effort and presented in a more clear and synthetic way. But the fundamental issue is that they were obtained with a method demonstrating the link between observed changes in the vital rates and observed changes in population performance. In fact, although Oppliger et al [13] did everything by the book (in this case the prospective analysis according to Caswell’ s book [17]) and even took extra care in demonstrating the vital rates (forcing functions of fitness) were effectively differentiated across an environmental gradient, this standard procedure was unable to synthesize information pinpointing the cause for the observed niche differentiation.

The first analysis by Oppliger et al. [13] performed 18 ANOVAs, three for each vital rate, setting temperature and species or temperature and location (within each species range) as factors. All this yielded one table with 14 rows and 14 columns, and one figure showing 6 graphics, each with 20 bars relative to 4 population × 5 temperature regimes. Then, Oppliger et al. [13] proceeded to the prospective analysis determining each population’s growth rate (λ) and their elasticities to the microstages’ vital rates [17]. Again, this led to three ANOVA’s, one figure with 20 bars and another figure with eight plots each containing a line showing λ and five bars, each divided into six areas, all totalling 240 areas. The authors relied on this information overdose to sustain that the microstages of the two kelp species basically differentiate every vital rate and with a significant impact on fitness. This was an overstatement because, although Oppliger et al. [13] proved that vital rates changed significantly and also did λ, they never demonstrated which changes in the vital rates were effectively inducing changes in λ, and this is the fundamental issue. The more complex methodologies that have been developed aiming to achieve this, namely the perturbation analysis [17], the LTRE [17] and the retrospective analysis [18], all ignore interactions between variables corresponding to the higher order terms of the Taylor expansion that this work proved being fundamental for the accuracy of the estimates. In fact, when comparing the northern most to the southern most population, interactions between vital rates could even represent more than half of the changes in fitness and their 3^rd^ order or higher terms could well represent about 40% of these changes.

Testing 60 seaweeds from the North Atlantic, Breeman [9] found temperature limitations on reproduction to be one of the three main processes determining their geographic distribution. Bolton and Anderson [8] and Matson and Edwards [11] found competing kelp species being geographically limited by the diverging effects of temperature on microstages performance and particularly on sexual reproduction. Kelps are not the only examples of seaweeds segregating by differentially adapting their microstages to the environment. Haploid and diploid spores of red algae with isomorphic biphasic life cycles were also found to perform differently in response to the environment and in particular to temperature [39, 40, 41, 42]. Furthermore, such differential responses were suggested as drivers for a niche partition [39, 42, 43, 44] required for the stability and evolution of these life cycles [43].

Clearly, both species perform better around 15°C. Nevertheless, while *Lessonia berteroana* seemed to be at its optimum, 60% of *Lessonia spicata*‘s populations clearly were not. Unfortunately, with only three temperatures tested it was impossible to implement a more suited resolution as in Bolton and Anderson [8]. These authors determined *Ecklonia biruncinata* and *Ecklonia maxima* bounded from slightly below 10°C to slightly above 25°C, with optima around 20°C and diverging only about 3°C. The mixed temperature incubations revealed to be a waist of effort as they did not provide any useful information but brought noise to the data. This effort would have been better applied providing the required finer resolution to the tested temperature range. The mixed temperatures experimental design was inappropriate as developmental stages could succeeded at a pace faster than the three days it took to shift temperatures. Furthermore, the resistance to disturbances should be better related to the variances of vital rates and their respective fitness components whereas their resilience should be better related to their magnitude. Therefore, it is reasonable to expect that the northern species deals better with the warmer temperatures to which kelps are exposed either seasonally [37] or associated to El Niño disturbances [45].
